# Intra-genomic GC heterogeneity in sauropsids: evolutionary insights from cDNA mapping and GC_3_ profiling in snake

**DOI:** 10.1186/1471-2164-13-604

**Published:** 2012-11-09

**Authors:** Kazumi Matsubara, Shigehiro Kuraku, Hiroshi Tarui, Osamu Nishimura, Chizuko Nishida, Kiyokazu Agata, Yoshinori Kumazawa, Yoichi Matsuda

**Affiliations:** 1 Department of Information and Biological Sciences, Graduate School of Natural Sciences, Nagoya City University, 1 Yamanohata, Mizuho-cho, Mizuho-ku, Nagoya, Aichi 467-8501, Japan; 2 Department of Biology, University of Konstanz, Universitaetsstrasse 10, Konstanz, 78457, Germany; 3 Genome Resource and Analysis Unit, RIKEN Center for Developmental Biology, 2-2-3 Minatojima-minamimachi, Chuo-ku, Kobe, Hyogo, 650–0047, Japan; 4 Laboratory for Molecular Developmental Biology, Department of Biophysics, Graduate School of Science, Kyoto University, Kitashirakawa-Oiwake, Sakyo-ku, Kyoto, Kyoto, 606-8502, Japan; 5 Department of Natural History Sciences, Faculty of Science, Hokkaido University, Kita 10 Nishi 8, Kita-ku, Sapporo, Hokkaido, 060-0810, Japan; 6 Laboratory of Animal Genetics, Department of Applied Molecular Biosciences, Graduate School of Bioagricultural Sciences, Nagoya University, Furo-cho, Chikusa-ku, Nagoya, Aichi, 464-8601, Japan; 7 Present address: Institute for Applied Ecology, University of Canberra, Canberra, ACT 2601, Australia; 8 Present address: Omics Science Center, Yokohama Institute RIKEN, 1-7-22 Suehiro-cho, Tsurumi-ku, Yokohama, Kanagawa, 230-0045, Japan

**Keywords:** GC-content, Lepidosauria, Snake, Macrochromosome, Microchromosome

## Abstract

**Background:**

Extant sauropsids (reptiles and birds) are divided into two major lineages, the lineage of Testudines (turtles) and Archosauria (crocodilians and birds) and the lineage of Lepidosauria (tuatara, lizards, worm lizards and snakes). Karyotypes of these sauropsidan groups generally consist of macrochromosomes and microchromosomes. In chicken, microchromosomes exhibit a higher GC-content than macrochromosomes. To examine the pattern of intra-genomic GC heterogeneity in lepidosaurian genomes, we constructed a cytogenetic map of the Japanese four-striped rat snake (*Elaphe quadrivirgata*) with 183 cDNA clones by fluorescence *in situ* hybridization, and examined the correlation between the GC-content of exonic third codon positions (GC_3_) of the genes and the size of chromosomes on which the genes were localized.

**Results:**

Although GC_3_ distribution of snake genes was relatively homogeneous compared with those of the other amniotes, microchromosomal genes showed significantly higher GC_3_ than macrochromosomal genes as in chicken. Our snake cytogenetic map also identified several conserved segments between the snake macrochromosomes and the chicken microchromosomes. Cross-species comparisons revealed that GC_3_ of most snake orthologs in such macrochromosomal segments were GC-poor (GC_3_ < 50%) whereas those of chicken orthologs in microchromosomes were relatively GC-rich (GC_3_ ≥ 50%).

**Conclusion:**

Our results suggest that the chromosome size-dependent GC heterogeneity had already occurred before the lepidosaur-archosaur split, 275 million years ago. This character was probably present in the common ancestor of lepidosaurs and but lost in the lineage leading to *Anolis* during the diversification of lepidosaurs. We also identified several genes whose GC-content might have been influenced by the size of the chromosomes on which they were harbored over the course of sauropsid evolution.

## Background

Molecular phylogenetic analyses have suggested that extant sauropsids (reptiles and birds) are divided into two major groups, the lineage of Testudines (turtles) and Archosauria (crocodilians and birds) and the lineage of Lepidosauria (tuatara, lizards, worm lizards and snakes) although phylogenetic position of Testudines is still debatable [1-7]. The divergence time between the two lineages has been estimated at around 275 million years [3,7-9]. Most sauropsidan species have karyotypes consisting of macrochromosomes and microchromosomes, as for birds [10-17], except for crocodilian species, whose karyotypes contain no microchromosomes [18,19].

Whole genome sequencing of chicken revealed that the overall GC-content of chromosomes increases as chromosomal size decreases, that is, microchromosomes exhibit a higher GC-content than macrochromosomes [20,21]. In a compositional map of GC-content constructed by 100-kb window analysis for the chicken whole genome sequence, most microchromosomes were occupied by GC-rich DNA segments, whereas GC-poor segments were more common in macrochromosomes [22]. The differences of other features such as gene density, distribution in interphase nuclei and rate of nucleotide divergence were also identified between the two chromosomal groups of birds [23-27].

Reptiles are crucial taxon for tracking genome evolution in amniotes [21,28,29]. Intra-genomic GC heterogeneity has been found in reptiles by calculating GC-content in exonic third positions (GC_3_) [21,30-33]. Although the use of GC_3_ as a proxy for genomic GC-content has been controversial [34], it is known that GC_3_ generally reflects the local GC-content of the introns and flanking regions of a gene [21,35-37]. Chojnowski *et al*. [32] analyzed the GC_3_ of more than 6,000 ESTs in the American alligator (*Alligator mississippiensis*) and suggested that the alligator genome has a certain level of GC heterogeneity. They also examined the isochore structure of the red-eared slider turtle **(***Trachemys scripta***)** and suggested that the isochore structure of the turtle is intermediate between that of a frog and the GC-rich isochore structures of archosaurs and mammals [33]. However, the chromosomal distribution of the GC heterogeneity has not been fully investigated in reptiles.

We previously constructed a cytogenetic map with 90 cDNA clones for the Chinese soft-shelled turtle (*Pelodiscus sinensis*), which revealed that the chromosomes have been highly conserved between the turtle and chicken, with the six largest chromosomes being almost equivalent to each other [38]. GC_3_ of the mapped genes showed a heterogeneous distribution, and orthologs exhibited similar GC_3_ levels between the turtle, chicken and human, suggesting that the intra-genome GC heterogeneity had already occurred in the last common ancestor of extant amniotes [21]. Furthermore, our results suggested that the turtle microchromosomes tend to contain more GC-rich genes than GC-poor genes, as in chicken [21].

The green anole lizard (*Anolis carolinensis*) is the first reptilian species for which whole genomic sequence has been released [39]. *Anolis* has a homogeneous genome composition compared with other amniotes [37,39] and, unlike chicken, the GC-content is similar between macro- and microchromosomes [39]. However, it remains unknown whether these genomic characteristics are common to other lepidosaurs or not. Snake karyotypes have been highly conserved within the group, and the usual diploid number is 2*n* = 36, consisting of eight pairs of macrochromosomes and 10 pairs of microchromosomes [10,40,41]. The chromosome number is largely different from the chicken karyotype (2*n*=78) because of the remarkable difference in the number of microchromosomes. The snake therefore provides an ideal system for exploring changes in GC-content between macro- and microchromosomes over the course of sauropsid evolution.

Previously we constructed a cytogenetic map with 109 cDNA clones for the Japanese four-striped rat snake, *Elaphe quadrivirgata* (Serpentes, Colubridae) [38,42]. In this study, we have extended cDNA-based chromosome mapping of the snake genes and consequently constructed a cytogenetic map with a total of 183 genes. We compared GC_3_ of the mapped snake genes with GC_3_ of their orthologs of chicken, green anole lizard, Chinese soft-shelled turtle, human and *Xenopus tropicalis*. This highlighted the chromosome size-dependent GC heterogeneity in the snake genome and the shift of GC-content possibly caused by chromosome rearrangements during sauropsid evolution.

## Methods

### Selection of EST clones

A cytogenetic map with 109 cDNA clones was constructed in our previous study [33,36]. In the present study, we searched the snake EST clones isolated from the cDNA library constructed from brain [38], selected clones with significant similarity (E-value < 2e^-35^) to human and/or chicken genes in BLASTX [43], and used them for chromosome mapping (Additional file 1 and Additional file 2).

### Orthology assessment

We rigorously confirmed orthologies of the snake sequences to their homologs of other vertebrates by constructing molecular phylogenetic trees with the neighbor-joining method [44] using XCED in which the alignment algorithm MAFFT is implemented [45] and with the maximum-likelihood method using PhyML [46]. Sequence IDs of orthologs in six species (*Anolis carolinensis*, *Gallus gallus*, *Pelodiscus sinensis*, *Homo sapiens*, *Mus musculus* and *Xenopus tropicalis*) are included in Additional file 2. When multiple more than one potential ortholog was detected for a snake gene, we used the sequence with the greatest similarity to snake for cross-species comparison of GC-content.

### Chromosome preparation and fluorescence in situ hybridization

Cell culture, preparation of R-banded chromosomes and fluorescence *in situ* hybridization (FISH) were performed as described previously [38,47]. Fibroblast cells derived from lung tissues of the Japanese four-striped rat snake were cultured and used for chromosome preparations. DNA probes were labeled by nick translation with biotin-16-dUTP (Roche Diagnostics). The hybridized cDNA probes were reacted with goat anti-biotin antibodies (Vector Laboratories), and then stained with Alexa488-labeled donkey anti-goat IgG (Molecular Probes).

### Calculation of GC-content

GC_3_ and GC-content at fourfold degenerate sites (GC_4_) were calculated using an original Perl script with the Bioperl module [48]. The calculation was automatically processed on the basis of the open reading frame identified by a pairwise alignment between a translated nucleotide sequence and amino acid sequences of orthologs using BLASTX [43]. When multiple alternative splicing variants were found for one gene, we used only the one that had the longest stretch of sequence homology with its orthologs of other species. We arbitrarily classified genes into GC-rich (GC_3_ ≥ 50%) and GC-poor (GC_3_ < 50%) genes based on the GC_3_.

### Identification of orthologous sequences in the Burmese python

We conducted nucleotide BLAST for whole genome shotgun sequence of Burmese python, *Python molurus bivittatus*[49] using rat snake ESTs as queries. We selected python sequences (consisting of exons, introns and flanking regions) that exhibited high similarities for rat snake ESTs (Additional file 3). We deduced the non-coding regions and the protein coding regions within each python genomic region using Wise2 program [50]. We then calculated GC-content of non-coding regions and GC_3_ of coding regions.

### Gene location in sequenced genomes

Chromosome locations of chicken, human and mouse orthologs were retrieved from Ensembl (version 56) [51] and NCBI Entrez Gene [52]. In this study, we categorized chicken chromosomes 1–8, Z and W as macrochromosomes, and the remaining chromosomes as microchromosomes. Recently the second version of the genome assembly and annotation of green anole lizard (*Anolis carolinensis*) was released in Ensembl (version 61) [51]. However, locations of about half of all *A. carolinensis* genes are at present annotated only at the scaffold level. We therefore did not compare the chromosome locations of orthologs between the snake and green anole lizard.

### Comparison of gene characteristics between chicken macrochromosomes and microchromosomes

We classified the chicken genes into two groups, macrochromosomal genes and microchromosomal genes, and examined the over- and under-representation of gene functions between the two groups by FatiGO [53]. FatiGO detects over-represented functional categories of Gene Ontology (GO), KEGG pathway, InterPro motif and Swissprot in either group between two gene lists using Fisher’s exact test. The Ensembl IDs of all chicken genes whose chromosome locations are known were downloaded and used for comparison by FatiGO.

## Results

### Cytogenetic map of the Japanese four-striped rat snake

**Figure 1:**
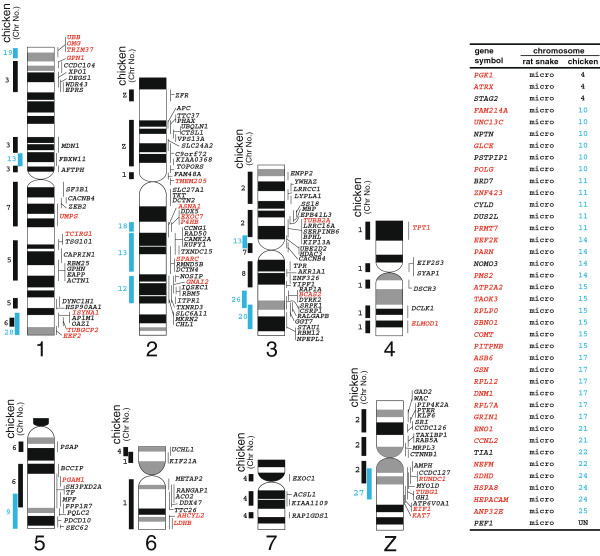
**Cytogenetic maps of macrochromosomes and a list of genes mapped to microchromosomes in *E. quadrivirgata*. **The chromosome locations of the genes are shown to the right of the rat snake chromosomes. The ideogram shows G-banded patterns. GC-rich (GC_3_ ≥ 50%) and GC-poor (GC_3 _< 50%) genes are shown in red and black, respectively. Homologous chicken chromosomes and their chromosome numbers (chicken Chr No.) are indicated to the left of the snake chromosomes. The inset table lists the genes mapped to snake microchromosomes and chromosome locations of their chicken orthologs are also given in the table. Chicken macrochromosomes (1–8 and Z) and microchromosomes (9–15, 17–22, 24–28) are distinguished by using different color, black and blue. ‘Un’ stands for unknown. The gene names are updated from our previous papers [38,42], using the latest Ensembl build (v 68). The chromosome locations of the two genes, *GNAI2 *(BW999984) and *P4HB *(BW999985), were changed from our previous study [37] by reexamination of FISH. Chromosome locations of chicken orthologs are also updated according to databases.

Eighty-three cDNA clones were newly mapped to the snake chromosomes, and finally a cytogenetic map with a total of 183 genes was constructed in this study (Figure 1, Additional file 1 and Additional file 2). The nucleotide sequences of the newly mapped EST clones were deposited in GenBank under the accession numbers FS942043-FS942125.

Of the 183 genes, 144 genes were mapped to macrochromosomes (chromosomes 1–7, Z and W chromosomes), and the others were mapped to microchromosomes (Figure 1). Twenty-nine segments in the snake chromosomes 1–7 and the Z chromosome were conserved between the snake and chicken. Most of them had a one to one correspondence to a particular region of chicken chromosomes. However, chromosomal homology for each of chicken chromosomes 1, 2, 4, 6 and 7 was found on more than two snake macrochromosomes, indicating that some inter-chromosomal rearrangements occurred between the snake and chicken macrochromosomes (Figure 1).

Chicken orthologs of 36 snake microchromosomal genes were located on chicken microchromosomes (chromosomes 10, 11, 14, 15, 17, 21, 22, 24 and 25). *PGK1*, *ATRX* and *STAG2* genes on the snake microchromosomes are localized to the short arm of chicken chromosome 4, which was derived from a microchromosome fused with acrocentric chromosome 4 of the avian ancestor [16,17,54]. Since all the snake microchromosomes corresponded to avian microchromosomes, they have likely been retained from the ancestral karyotype of extant sauropsids without dynamic chromosome rearrangements.

Linkage homologies with chicken microchromosomes 9, 10, 12, 13, 18–20 and 26–28 were found on the snake macrochromosomes (Figure 1). For example, chicken chromosomes 19 and 28 were homologous to the distal segments of the short and long arms of snake chromosome 1, respectively. These results confirmed our previous assumption [42] that the large differences of chromosome numbers between the snakes (2*n* = 36) and chicken (2*n* = 78) resulted from frequent chromosome rearrangements containing fusions between macro- and microchromosomes and also between microchromosomes in the lepidosaurian lineage. An alternative explanation is that fissions of macrochromosomes, which increase microchromosomes, may also have occurred in the lineage leading to birds.

### Intra-genomic heterogeneity of GC_3_ in snake

**Figure 2:**
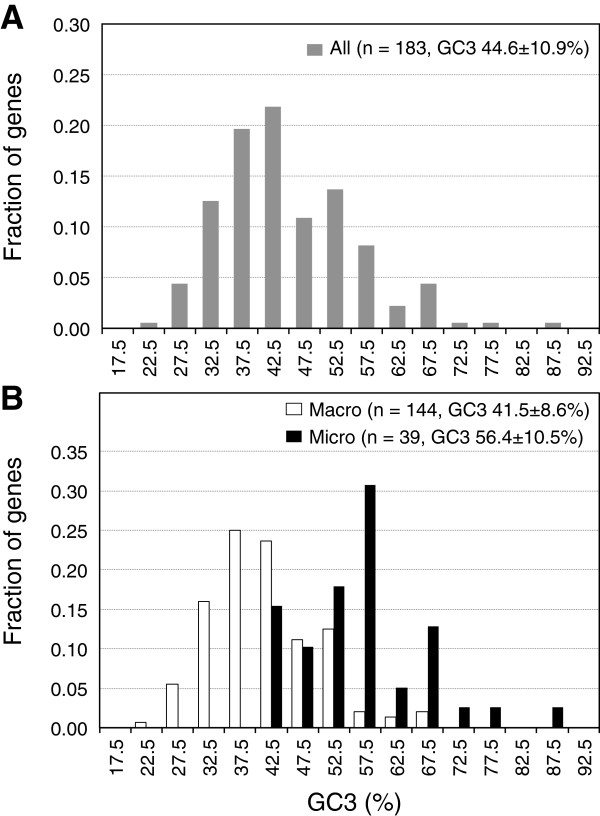
**GC**_**3 **_**distribution of the snake genes. **Histograms show frequency distributions of GC_3 _for all genes (**A**), and for macrochromosomal: open columns and microchromosomal genes: black columns (**B**).

We calculated the GC_3_ for deduced protein-coding regions of the 183 snake genes (Additional file 2). The average and standard deviation of GC_3_ of the snake genes were 44.6% and 10.9% (Figure 2A) and a similar result was obtained when GC_4_ was analyzed (data not shown). The averages of chicken, the soft-shelled turtle and human orthologs were 51.4 ± 13.0% (mean ± standard deviation), 46.5 ± 12.6% and 53.9 ± 16.8%, respectively (Additional file 4). The average and standard deviation of GC_3_ of snake genes were thus somewhat smaller than those of the other amniotes. The distribution of GC_3_ of the snake genes was also relatively narrow (Figure 2A). In order to examine bimodality of the distribution, we compared the fit of a “Gaussian model” and “sum of two Gaussians model” by Extra sum-of-squares F test and Akaike’s Information Criterion implemented in GraphPad Prism (GraphPad Software). As a result, “sum of two Gaussians model” showed a better fit in both test (p < 0.005 in Extra sum-of-squares F test and 73.28% probability in Akaike’s Information Criterion). This suggests that GC_3_ of the snake genes exhibits a bimodal distribution.

GC_3_ was compared between macro- (n = 144) and microchromosomal genes (n = 39) to test for the presence of chromosome size-dependent GC_3_ heterogeneity in the snake genome (Figure 2B). The average GC_3_ was 41.5 ± 8.6% (mean ± standard deviation) and 56.4 ± 10.5% for macrochromosomal and microchromosomal genes, respectively. The average GC_3_ of microchromosomal genes was thus significantly higher than for macrochromosomal genes (Mann-Whitney’s *U*-test, *P* < 0.01). 74.4% (29 out of 39 genes) of the microchromosomal genes were GC-rich, whereas 81.9% (118 out of 144 genes) of the macrochromosomal genes were GC-poor (Table 1).

**Table 1:** Relationships between GC-content and chromosome location of the snake genes

	**GC-rich gene (GC**_**3**_**≥50)**	**GC-poor gene (GC**_**3**_**≤50)**	**Total**
Macrochromosomal gene	26	118	144
Microchromosomal gene	29	10	39
Total	55	128	183

GC-rich isochores are known to have a clear association with R-bands (and particularly T bands) in mammals and birds [55-58]. In R-banded metaphases of rat snake, most microchromosomes showed R-positive bands (Additional file 5). However, R-positive bands also observed on most macrochromosomal regions. Thus, there was no clear correlation between R-band and GC_3_ of the mapped genes in rat snake.

### Correlation between GC_3_ and GC-content of non-coding regions in snakes

**Figure 3:**
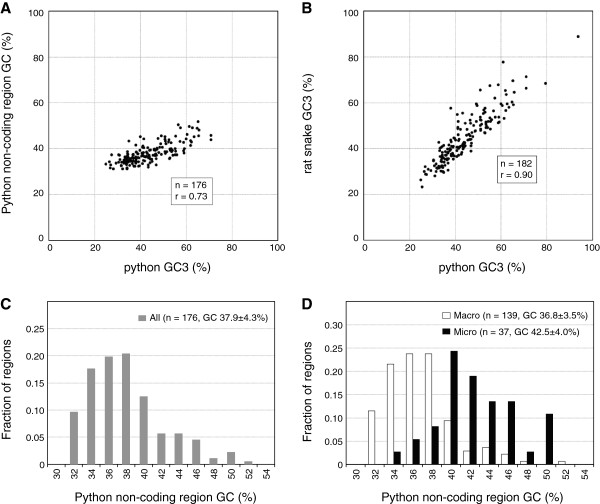
**Comparison of GC**_**3 **_**and GC-content of non-coding regions, and distribution of GC-content of non-coding regions. **Two-dimensional plots show the correlation between the GC_3 _of genes and the GC-content of non-coding regions surrounding the genes in the Burmese python (**A**) and the correlation of the GC_3 _of the orthologs between the python and the rat snake (**B**). Frequency distributions of GC-content are shown as histograms for all the python non-coding regions (**C**), for the macrochromosomal regions (open columns) and the microchromosomal regions (black columns) (**D**).

Snake karyotypes are highly conserved among the species [10,40,41], and the Japanese four-striped rat snake (*Elaphe quadrivirgata*) and the Burmese python (*Python molurus bivittatus*) have the same chromosome composition [42]. We compared the GC_3_ with the GC-content of non-coding regions in the python to examine whether snake GC_3_ reflects the local genomic GC-content (Figure 3A and Additional file 3). High positive correlation was found between GC_3_ and GC-content of non-coding regions (n = 176) (Spearman’s rank correlation, r = 0.73, *P* < 0.01). In order to consider the differences of base composition among species [36,59,60], we also compared GC_3_ of orthologs between the python and the rat snake (n = 182) (Figure 3B and Additional file 3). Strong correlation was found (r = 0.90; *P* < 0.01). These results suggest that the two species have similar genomic compositions and snake GC_3_ reflects the local genomic GC-content.

The GC-content of the python non-coding regions showed a narrow distribution with a low average (37.9 ± 4.3%, mean ± standard deviation) in contrast to GC_3_ (Figure 3A and C). We divided the python non-coding regions into macrochromosomal (n = 139) and microchromosomal regions (n = 37) on the postulate that the chromosome locations of all orthologs were conserved between the rat snake and the python, and compared the GC-content between the two chromosomal groups (Figure 3D). The average GC-content of microchromosomal regions (42.5 ± 4.0%, mean ± standard deviation) were significantly higher than those of macrochromosomal regions (36.8 ± 3.5%) (Mann-Whitney’s *U*-test, *P* < 0.01).

### Cross-species comparison of GC_3_ between orthologs of amniotes

We examined the frequency distribution of GC_3_ of orthologs in green anole lizard, chicken, Chinese soft-shelled turtle, human, mouse and an amphibian species *X. tropicalis* (Additional file 4). In previous study, we analyzed the frequency distribution of GC_3_ with massive genes in these species except for anole lizard. The distribution patterns of GC_3_ of this study were similar to those in Figure 2 of our previous study [21] although the gene set of this study contains a somewhat higher proportion of GC-poor genes. Thus we thought that GC_3_ of the gene set of this study could be used as a representative of GC_3_ of the whole genes in each species.

**Figure 4:**
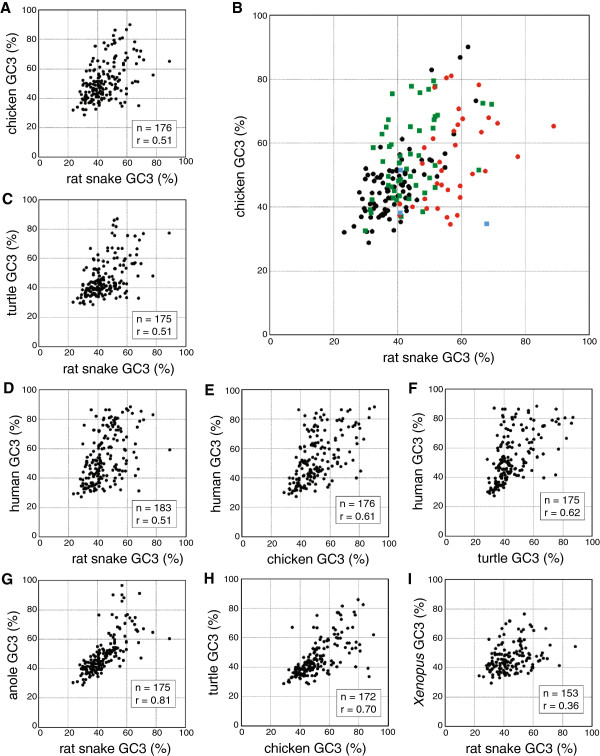
**Cross-species comparison of GC**_**3 **_**between orthologs. **Two-dimensional plots of GC_3 _for orthologous gene pairs are shown for rat snake-chicken (**A**, **B**), rat snake-Chinese soft-shelled turtle (**C**), rat snake-human (**D**), chicken-human (**E**), Chinese soft-shelled turtle-human (**F**), rat snake-green anole lizard (**G**), chicken-Chinese soft-shelled turtle (**H**) and rat snake-*Xenopus tropicalis* (**I**). At (B), orthologs are divided into the following four categories as described in text: 1) genes located on macrochromosomes in both species (black dots), 2) genes on microchromosomes in both species (red dots), 3) genes on macrochromosomes in the snake and on microchromosomes in the chicken (green dots), and 4) genes on microchromosomes in the snake and on macrochromosomes in the chicken (blue dots, i.e., *PGK1*, *ATRX* and *STAG2*; see Figure 1).

Then, to examine whether orthologs exhibit similar levels of GC_3_ between species, we compared the GC_3_ of the snake genes with orthologs in other species (Figure 4 and Additional file 2). Moderate positive correlations were found for snake-chicken (n = 176), snake-turtle (n = 175) and snake-human (n = 183) comparisons with correlation coefficients r = 0.51 (Spearman’s rank correlation, *P* < 0.01). Similarly, moderate positive correlations were found for chicken-human (n = 176) (r = 0.61; *P* < 0.01) and turtle-human (n = 175) (r = 0.62; *P* < 0.01), respectively. Much higher correlation was found for snake-anole lizard (n = 175) (r = 0.81; *P* < 0.01) and chicken-turtle (n = 172) (r = 0.70; *P* < 0.01), respectively. In contrast, only weak correlation was found for snake-*X. tropicalis* (n = 153) (r = 0.36; *P* < 0.01).

**Figure 5:**
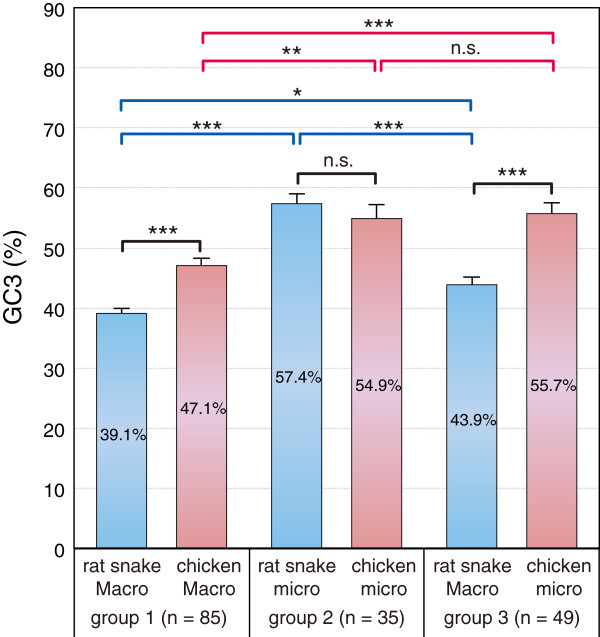
**Comparison of the GC**_**3 **_**between the species and among the gene groups. **Histograms show mean ± SEM of GC_3_. The rat snake and chicken orthologs are grouped as described in text: group 1) genes located on macrochromosomes in both species, group 2) genes located on microchromosomes in both species, and group 3) genes located on macrochromosomes in the snake and on microchromosomes in the chicken. Because only three genes were classified into the 4th group, indicated by blue dots in Figure 4B, the GC_3 _of this group was not compared statistically and thus omitted from this figure. Horizontal lines above bars show differences. Significances for comparison between species (black lines) and for comparison between groups (blue and red lines) are examined by Mann-Whitney’s *U*-test and the Kruskal-Wallis test with Dunn's post test, respectively. *P<0.05, **P<0.01, ***P<0.001 and n.s., not significant.

For the snake-chicken pair, we also compared the GC_3_ of orthologs with four categories: 1) genes located on macrochromosomes in both species (n = 85), 2) genes located on microchromosomes in both species (n = 35), 3) genes located on snake macrochromosomes and on chicken microchromosomes (n = 49), and 4) genes located on snake microchromosomes and on chicken macrochromosomes (n = 3) (Figure 4B). Four orthologs whose chromosome locations are unknown were excluded from this comparison. The average GC_3_ of the snake and chicken orthologs were 39.1 ± 8.0% (mean ± standard deviation) and 47.1 ± 10.9% in the 1st group, 57.4 ± 9.8% and 54.9 ± 13.7% in the 2nd group, and 43.9 ± 8.7% and 55.7 ± 12.8% in the 3rd group, respectively (Figure 5). We were not able to compare the GC_3_ statistically in the 4th group because only three genes were classified into this group.

### Comparison of gene functions between macro- and microchromosomes

**Figure 6:**
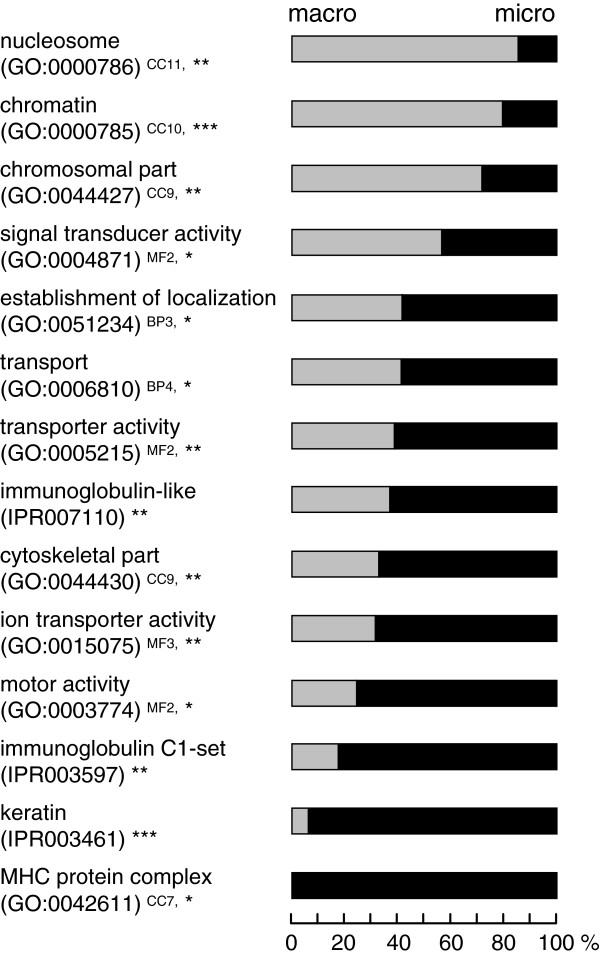
**Statistically over-represented Gene Ontology or InterPro categories between chicken macrochromosomal and microchromosomal genes. **Over-representation of gene categories was examined between the genes on chicken macrochromosomes (chromosomes 1–8, Z and W) and microchromosomes (chromosomes 9–28). The proportion of the macro- and microchromosomal genes is shown in each over-represented category of Gene Ontology (GO) or InterPro (IPR). The classifications of GO categories are indicated by superscripts: CC, cellular component; MF, molecular function; BP, biological process; Numbers indicate the levels of GO hierarchy. Significance for each category is assessed with Fisher’s exact test; *P<0.05, **P<0.01 and ***P<0.001.

To examine the relationships between gene functions and chromosome size-dependent GC bias in chicken, we assessed over-representation of functional categories of Gene Ontology (GO), KEGG pathway, InterPro motif and Swissprot in either of two gene groups: the 10,053 genes on chicken macrochromosomes (namely, chromosomes 1–8, Z and W) and 6,297 genes on chicken microchromosomes (namely, chromosomes 9–28). Chromosomes 29–31 and 33–38 were excluded from analysis because of their absence from the assembled genome. No genes have been assigned to chromosome 32, although the genome sequence was anchored to this chromosome. Fourteen GO terms or InterPro domains were over-represented in either macrochromosomal genes or microchromosomal genes (Figure 6 and Additional file 6: Table S1). Over-representation of "chromatin" (GO:0000785), "nucleosome" (GO:0000786) and "chromosomal part" (GO:0044427) on macrochromosomes largely depended on the abundance of members of the histone gene family on chromosome 1 (Additional file 6: Table S9, S11 and S12). Over-representation of "immunoglobulin C1-set" (IPR003597) and "MHC protein complex" (GO:0042611) in microchromosomes came from clustering of many immune genes on chromosome 16 (Additional file 6: Table S8 and S14). Similarly, over-representation of "keratin" (IPR003461) was due to the abundance of keratin-like gene family members on chromosomes 25 and 27 (Additional file 6: Table S13). Over-representation of the other eight categories in either macrochromosomes or microchromosomes was not attributable to an excess of genes on particular chromosomes (Additional file 6: Table S1-S7, S10 and S15).

## Discussion

### Chromosome size-dependent GC heterogeneity is a pan-Sauropsida characteristic

We constructed a cytogenetic map of the Japanese four-striped rat snake, which contained 183 genes. Our cytogenetic map covered most macrochromosomal regions and at least one gene was mapped to each microchromosome on the basis of the homologies with chicken chromosomes. Our map also showed linkage homologies with most chicken chromosomes, namely 1–15, 17–22, 24–28 and Z. These results make it possible to infer the global GC heterogeneity of the snake genome and the shift of GC-content caused by chromosome rearrangements during sauropsid evolution.

We calculated GC_3_ to investigate the intra-genomic GC heterogeneity in the rat snake. The GC_3_ of the snake genes exhibited a bimodal distribution (Figure 2A). Such a bimodal distribution of GC_3_ was also observed in the genomes of the Chinese soft-shelled turtle (*P. sinensis*), chicken and non-rodent mammals [21]. This result suggests that the GC_3_ heterogeneity is a common feature of amniote genomes. However, the standard deviation of GC_3_ in snake genes was somewhat lower than that of other amniotes [21], and GC-content of python non-coding regions also showed a narrow distribution, as observed in the green anole lizard [37]. Thus the heterogeneities of base composition have probably decreased in lepidosaurian lineages over evolutionary time.

Although the standard deviation of GC_3_ of snake genes was relatively small, our results suggest that snake microchromosomes contain a higher proportion of GC-rich genes than macrochromosomes, as observed in both the Chinese soft-shelled turtle and chicken [21]. Recently, chromosome size-dependent GC heterogeneity was also identified in the red-eared slider turtle (*Trachemys scripta elegans*) and the Nile crocodile (*Crocodylus niloticus*) using chromosome flow sorting technique [61]. Chromosome size-dependent GC heterogeneity therefore seems to be a widespread characteristic in sauropsids whose karyotypes consist of macrochromosomes and microchromosomes, and possibly originated in the common ancestor of sauropsids. Interestingly, the green anole lizard, whose karyotype consists of 6 pairs of macrochromosomes and 12 pairs of microchromosomes [39,62], does not show such marked biases in GC-content between macro- and microchromosomes. This suggests that the chromosome size-dependent GC heterogeneity has disappeared in the lineage leading to the anole lizard. Lepidosauria is a species-rich group consisting of about 8,000 species, and the karyotypes are also diversified within the group. Further investigation in various lepidosaurian species may help clarify the relationship between GC-content and the karyotype.

### Disparity between lepidosaurs and the turtle-archosaurs

The correlation coefficient of GC_3_ between the rat snake and chicken orthologs are lower than that between the Chinese soft-shelled turtle and chicken (Figure 4). One explanation for the lower correlation is that the phylogenetic distance between the snake and chicken is larger than between the turtle and chicken. However, the divergence time between turtles and birds is estimated to be more than 231 million years, which is not largely different from the time of the lepidosauria-archosauria split, 275 million years ago [3,7-9]. Therefore we consider the effect of the large differences of karyotypes, especially the number of microchromosomes, between the snake and the other two species.

The chromosome numbers are largely different between the rat snake (2*n* = 36) and chicken (2*n* = 78). In contrast, the karyotype of the Chinese soft-shelled turtle, which consists of nine pairs of macrochromosomes and 24 pairs of microchromosomes (2*n* = 66), is very similar to the chicken karyotype [38]. Our previous study demonstrated by comparative gene mapping that the chromosomes have been highly conserved between chicken and the turtle [21,38]. Chicken microchromosomes were considered to extensively retain the ancestral linkage groups of genes [63], and Nakatani *et al.*[64] also suggested that many chicken microchromosomes (i.e., chromosomes 11, 15, 19, 20, 21, 22, 23, 24, 27 and 28) have one-to-one correspondence to ancestral proto-chromosomes of the gnathostome ancestor [64]. These results lead us to infer that chromosome rearrangements have occurred more frequently in the snake lineage than in the chicken lineage, that is, chromosome number has been reduced by frequent chromosome fusions between macro- and microchromosomes and also between microchromosomes in the snake lineage.

**Figure 7:**
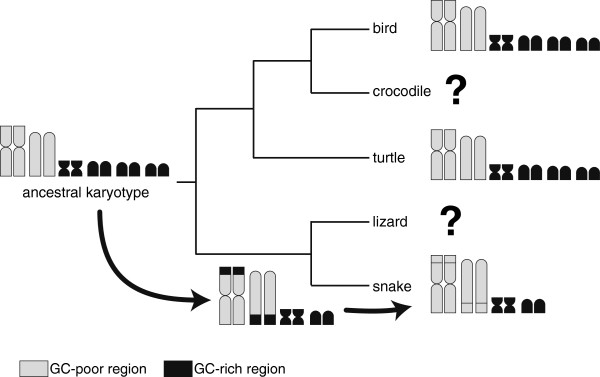
**Schematic representation of the process of chromosome rearrangements and the change of GC-content in sauropsids. **Phylogenetic relationships of major groups of sauropsids are based on molecular phylogenetic data [3,6,7]. This model postulates that chromosome size-dependent GC heterogeneity occurred in the ancestral karyotype. This feature is retained almost intact in the lineages of birds and turtles where interchromosomal rearrangements are infrequent. Some chromosome fusions have occurred in the lepidosaurian lineage and GC_3_ of genes has been altered according to the size of the chromosome on which they are borne.

Eleven chromosome segments homologous to chicken microchromosomes were localized to the snake macrochromosomes in this study (Figure 1). The GC_3_ of the snake orthologs on these macrochromosomal segments were lower than those of their chicken orthologs on microchromosomes (green dots of Figure 4B and 5). For instance, 16 of 21 snake orthologs mapped on chromosome 2q, which is homologous to chicken chromosomes 18, 13 and 12, were GC-poor (GC_3_ < 50%), whereas 9 of 17 chicken orthologs on chicken chromosomes 18, 13 and 12 were GC-rich (GC_3_ ≥ 50%) (Additional file 2). These results suggest that changes in chromosome sizes caused the differences of GC_3_ levels between the chicken microchromosomal genes and their snake orthologs on macrochromosomal segments derived from the ancestral microchromosomes (Figure 7).

### Impact of chromosome fissions/fusions on GC-content

What mechanisms were involved in the changes of the GC-content of the genes after the fusion of microchromosomes into macrochromosomal complement? It has been suggested that the GC-content is primarily influenced by local recombination rates via GC-biased gene conversion [65,66]. Under this model, A or T is displaced by G or C through mismatch repair when an AT/GC heteroduplex is formed at recombining regions. Accordingly, AT/GC heterozygotes produce more GC than AT gametes, thus conferring predominance of GC alleles in frequently recombining regions.

Recombination rate is negatively correlated with the size of chromosome arms in human and chicken [20,67]. In chicken in particular, recombination rate per unit physical length is much higher in microchromosomes than in macrochromosomes [20]. Recombination rates per physical length are thus expected to be lower in the snake macrochromosomal segments derived from the ancestral microchromosomes than those in their homologous chicken microchromosomes. The chromosome size-dependent difference in the recombination rate thus seems to have caused the decrease of GC-content in the snake macrochromosomal genes derived from the microchromosomes of the common ancestor of sauropsids.

Empirical evidence of chromosome length-driven evolution of GC-content has been shown in marsupial and monotreme species [36]. In contrast, chromosome size-dependent GC heterogeneity was not clearly demonstrated in eutherian species, likely because frequent chromosome rearrangements have obscured the history of GC-content changes [36]. In many eutherian lineages, however, intra-chromosomal GC heterogeneity has been reported [21,36,68,69]. In chicken, intra-chromosomal GC heterogeneity is not as prominent as in eutherians [22], and there is no intra-chromosomal GC heterogeneity known in the anole genome [37,39]. Our approach could not demonstrate whether there was intra-chromosomal GC heterogeneity in snake genome because of the insufficient sequence data provided by cDNA sequencing. It is necessary to conduct whole genome sequencing and assemble the sequences into a chromosome scale in order to clarify the relationship between intra- and inter-chromosomal GC heterogeneity in the snake genomes.

When overall GC-content and recombination rates were compared among various vertebrate species, there was no clear correlation between GC-content and recombination rates [70]. For example, the recombination rate of chicken is about two times higher than that of zebra finch, although GC-content is almost equal between the two species [70,71]. Whereas rat snake microchromosomal genes show similar level of GC_3_ to chicken microchromosomal genes, GC_3_ of rat snake macrochromosomal genes is significantly lower than chicken macrochromosomal genes (Figure 5). Thus other factors might exert influences on overall genomic GC-content. In bacteria, genomic GC-content have been subject to natural selection but not to biased gene conversion [72]. Modes other than biased gene conversion were also proposed for evolution of genome composition in vertebrates [68]. Further consideration is therefore necessary for the evolution of overall genome composition in snakes.

### Any biological partitioning between macrochromosomes and microchromosomes?

Many literatures reported existing correlations between gene function and base compositions of the genes, the genomes and the promoter regions [73-75]. The difference of global GC-content between the macro- and microchromosomes may potentially cause the biased distribution of gene functions between the chromosomes: some proteins containing more amino acids for GC-rich codons due to functional constraints may be more advantageous in being encoded in microchromosomes than in macrochromosomes. To test this possibility, we investigated functional difference between chicken macro- and microchromosomal genes on the basis of the frequencies of appearance of functional categories (Figure 6). Over-representations found in six categories could be caused by clustering of particular gene families in short genomic stretches. These gene families may be less informative to test the hypothesis that genes are selected based on the size of chromosomes on which they are encoded. The over-representations of the other eight categories were independent from an excess of genes on particular chromosomes, implying that there could be some functional differences between macro- and microchromosomal genes.

In order to examine the relationship between the apparent localization of functional gene categories and the chromosome size-dependent GC-content, we investigated the GC-content of the gene sets assorted in the eight over-represented categories: GO:0004871, GO:0051234, GO:0006810, GO:0005215, IPR007110, GO:0044430, GO:0015075 and GO:0003774 (Additional file 6: Table S16). As shown in Figure 6 and Additional file 6: Table S1, seven of the eight categories were over-represented in microchromosomes. If most genes assorted in these GO or InterPro categories have high GC-content, it is likely that functional compartmentalization of genes is influenced by the difference of global GC-content between macro- and microchromosomes, i.e., some functionally categorized gene groups with high GC-content have been selectively preserved on microchromosomes. The average GC-content of the genes of the seven categories ranged from 43.9 to 48.1% and most of them were lower than the average of all chicken genes (47.4%) (Additional file 6: Table S16). In the other six categories (GO:0042611, GO:0044427, GO:0000785, GO:0000786, IPR003461 and IPR003597), there was no clear correlation between abundance in macro- or microchromosomes and GC-content. For an example, GO:0044427, GO:0000785 and GO:0000786 were over-represented in macrochromosomes, but their GC-contents were relatively high (Additional file 6: Table S1 and S16). These results imply that functional differences of genes did not correlate with the global difference of GC-content between macro- and microchromosomes. Further characterization of functional difference between macro- and microchromosomal genes, as well as its correlation with the general GC trend in chromosomal environments should await more extensive analyses using multiple species in future.

## Conclusion

In this study, we constructed a cytogenetic map with 183 genes in the Japanese four-striped rat snake, and calculated GC_3_ across all chromosomes. Our results revealed cytogenetic evidence that snake microchromosomal genes tend to have higher GC_3_ than macrochromosomal genes, as found in the chicken and the Chinese soft-shelled turtle, a feature apparently lost in the genome of anole lizard. By comparing GC_3_ of orthologs between snake and chicken, we show that the GC-content of genes is correlated with the size of chromosomes on which the genes are harbored. This chromosome size-dependent GC heterogeneity is particularly apparent in snake genes that have been translocated from microchromosomes to macrochromosomes since snakes and birds shared a common ancestor, some 275 million years. The addition of whole genome sequencing and karyotypes from wide variety of sauropsidan species will provide the fine-scale picture of timing and mode of GC shift accompanying karyotypic evolution in this important group of vertebrates.

## Abbreviations

cDNA: Complementary DNA; GC_3_: GC-content of exonic third codon positions; EST: Expressed sequence tag; FISH: Fluorescence *in situ* hybridization; GO: Gene Ontology.

## Competing interests

The authors declare that they have no competing interests.

## Authors' contributions

KM, SK, YK and YM designed the study. HT, ON and KA constructed the EST library. KM and CN conducted FISH mapping. KM and SK analyzed the data. KM, SK, YK and YM wrote the paper. All authors read and approved the manuscript.

## Supplementary Material

Additional file 1 **FISH mapping of cDNA clones in *Elaphe quadrivirgata*. **FISH mapping of six cDNA clones in *Elaphe quadrivirgata*. Click here for file

Additional file 2 **IDs and GC**_**3 **_**of 184 orthologous gene sets. **IDs and GC_3 _of 184 orthologous gene sets. Click here for file

Additional file 3 **IDs and GC-content of the python contigs that include the orthologs of the rat snake ESTs. **IDs and GC-content of the python contigs that include the orthologs of the rat snake ESTs. Click here for file

Additional file 4 **GC**_**3 **_**distribution of the orthologs in other vertebrates. **GC_3_ distribution of the orthologs in other vertebrates. Click here for file

Additional file 5 **R-banded karyotype of *Elaphe quadrivirgata. ***R-banded karyotype of *Elaphe quadrivirgata*. Click here for file

Additional file 6 **Table S1. **Functional categories over-represented in either gene group, chicken macrochromosomal genes or microchromosomal genes. **Table S2. **Chicken macrochromosomal and microchromosomal genes assoted into GO:0051234 and their chromosome locations and GC-contents. **Table S3. **Chicken macrochromosomal and microchromosomal genes assoted into GO:0006810 and their chromosome locations and GC-contents. **Table S4. **Chicken macrochromosomal and microchromosomal genes assoted into GO:0005215 and their chromosome locations and GC-contents. **Table S5.** Chicken macrochromosomal and microchromosomal genes assoted into GO:0003774 and their chromosome locations and GC-contents. **Table S6. **Chicken macrochromosomal and microchromosomal genes assoted into GO:0004871 and their chromosome locations and GC-contents. **Table S7. **Chicken macrochromosomal and microchromosomal genes assorted into GO:0015075 and their chromosome locations and GC-contents. **Table S8. **Chicken macrochromosomal and microchromosomal genes assorted into GO:0042611 and their chromosome locations and GC-contents. **Table S9.** Chicken macrochromosomal and microchromosomal genes assorted into GO:0044427 and their chromosome locations and GC-contents. **Table S10. **Chicken macrochromosomal and microchromosomal genes assorted into GO:0044430 and their chromosome locations and GC-contents. **Table S11. **Chicken macrochromosomal and microchromosomal genes assorted into GO:0000785 and their chromosome locations and GC-contents. **Table S12. **Chicken macrochromosomal and microchromosomal genes assorted into GO:0000786 and their chromosome locations and GC-contents. **Table S13.** Chicken macrochromosomal and microchromosomal genes assorted into IPR003461 and their chromosome locations and GC-contents. **Table S14. **Chicken macrochromosomal and microchromosomal genes assorted into IPR003597 and their chromosome locations and GC-contents. **Table S15. **Chicken macrochromosomal and microchromosomal genes assorted into IPR007110 and their chromosome locations and GC-contents. **Table S16. **The number of genes and mean of GC-content of macro- and microchromosomal genes assorted into each over-represented functional category. The detailed tables for statistically over-represented categories of Gene Ontology or InterPro between the chicken macrochromosomal and microchromosomal genes. Click here for file
