# Supplementary material for: Intra-genomic GC heterogeneity in sauropsids: evolutionary insights from cDNA mapping and GC3 profiling in snake
Source: BMC Genomics. 2012 Nov 9;13:604. doi: 10.1186/1471-2164-13-604 (PMC3549455; doi:10.1186/1471-2164-13-604)
Supplement: Additional file 1 — FISH mapping of cDNA clones in Elaphe quadrivirgata. FISH mapping of six cDNA clones in Elaphe quadrivirgata. [file 1471-2164-13-604-S1.pdf]

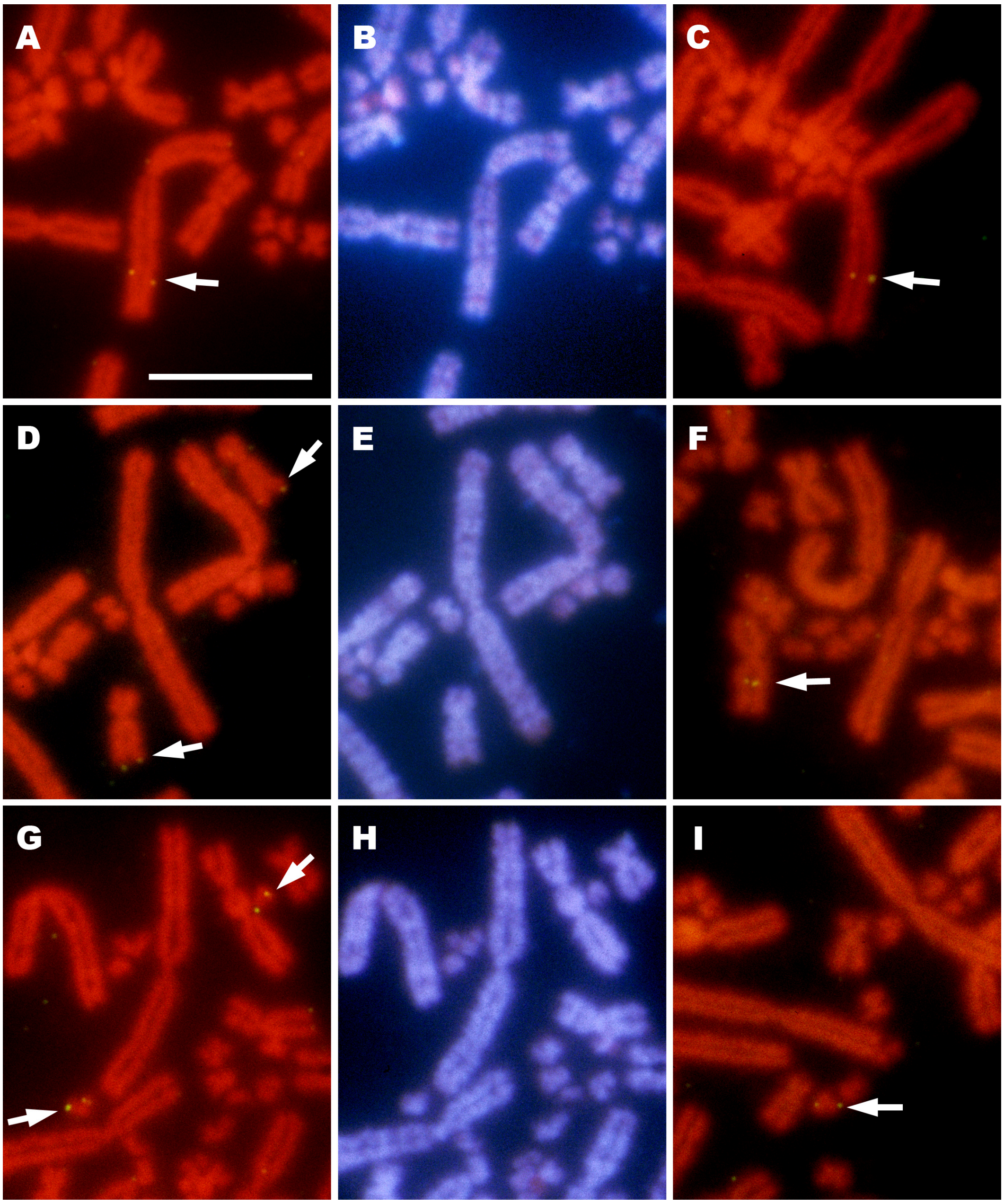

Additional file 1. FISH mapping of cDNA clones in *Elaphe quadrivirgata*. *CHL1* (A and B), *ACTN1* (C), *AHCYL2* (D and E) and *ATP6V0A1* (F) were mapped on chromosomes 2, 1, 6 and Z chromosome, respectively. *CCNL2* (G and H) and *STAG2* (I) were mapped on microchromosomes. Arrows indicate the hybridization signals. Hoechst-stained G-banded patterns of the same metaphase as in (A), (D) and (G) are shown in (B), (E) and (H), respectively. A scale bar indicates 10 μm. All microphotographs have the same magnification.
