# Supplementary material for: Intra-genomic GC heterogeneity in sauropsids: evolutionary insights from cDNA mapping and GC3 profiling in snake
Source: BMC Genomics. 2012 Nov 9;13:604. doi: 10.1186/1471-2164-13-604 (PMC3549455; doi:10.1186/1471-2164-13-604)
Supplement: Additional file 4 — GC3 distribution of the orthologs in other vertebrates. GC3 distribution of the orthologs in other vertebrates. [file 1471-2164-13-604-S4.pdf]

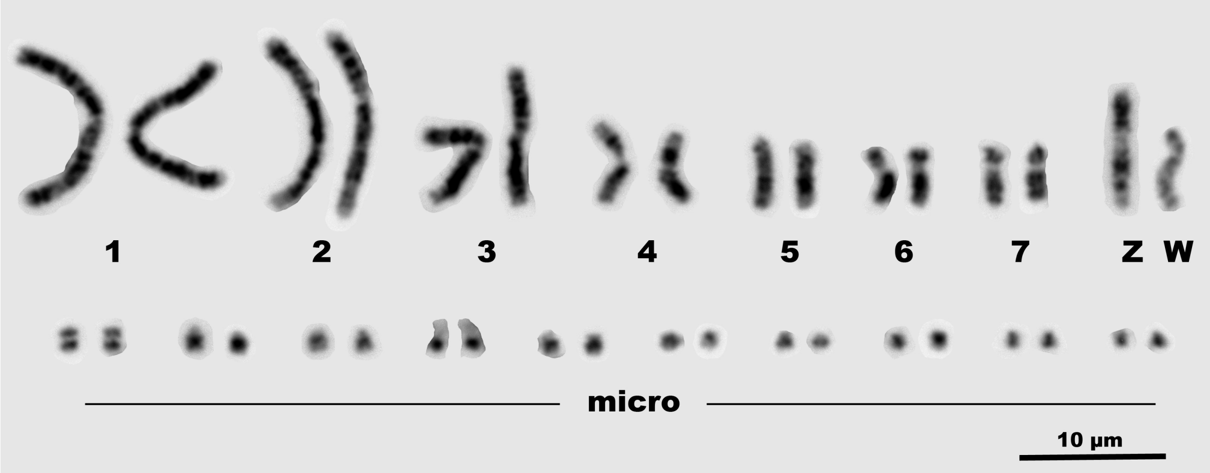

Additional file 5. R-banded karyotype of *Elaphe quadrivirgata*. R-banded chromosomes were stained with Giemsa and aligned according to the G-banded karyotype. Chromosome number of the Japanese four-striped rat snake (*Elaphe quadrivirgata*) is 2*n* = 36, consisting of eight pairs of macrochromosomes and 10 pairs of microchromosomes. A scale bar indicates 10 µm.
