# Supplementary material for: Intra-genomic GC heterogeneity in sauropsids: evolutionary insights from cDNA mapping and GC3 profiling in snake
Source: BMC Genomics. 2012 Nov 9;13:604. doi: 10.1186/1471-2164-13-604 (PMC3549455; doi:10.1186/1471-2164-13-604)
Supplement: Additional file 5 — R-banded karyotype of Elaphe quadrivirgata. R-banded karyotype of Elaphe quadrivirgata. [file 1471-2164-13-604-S5.pdf]

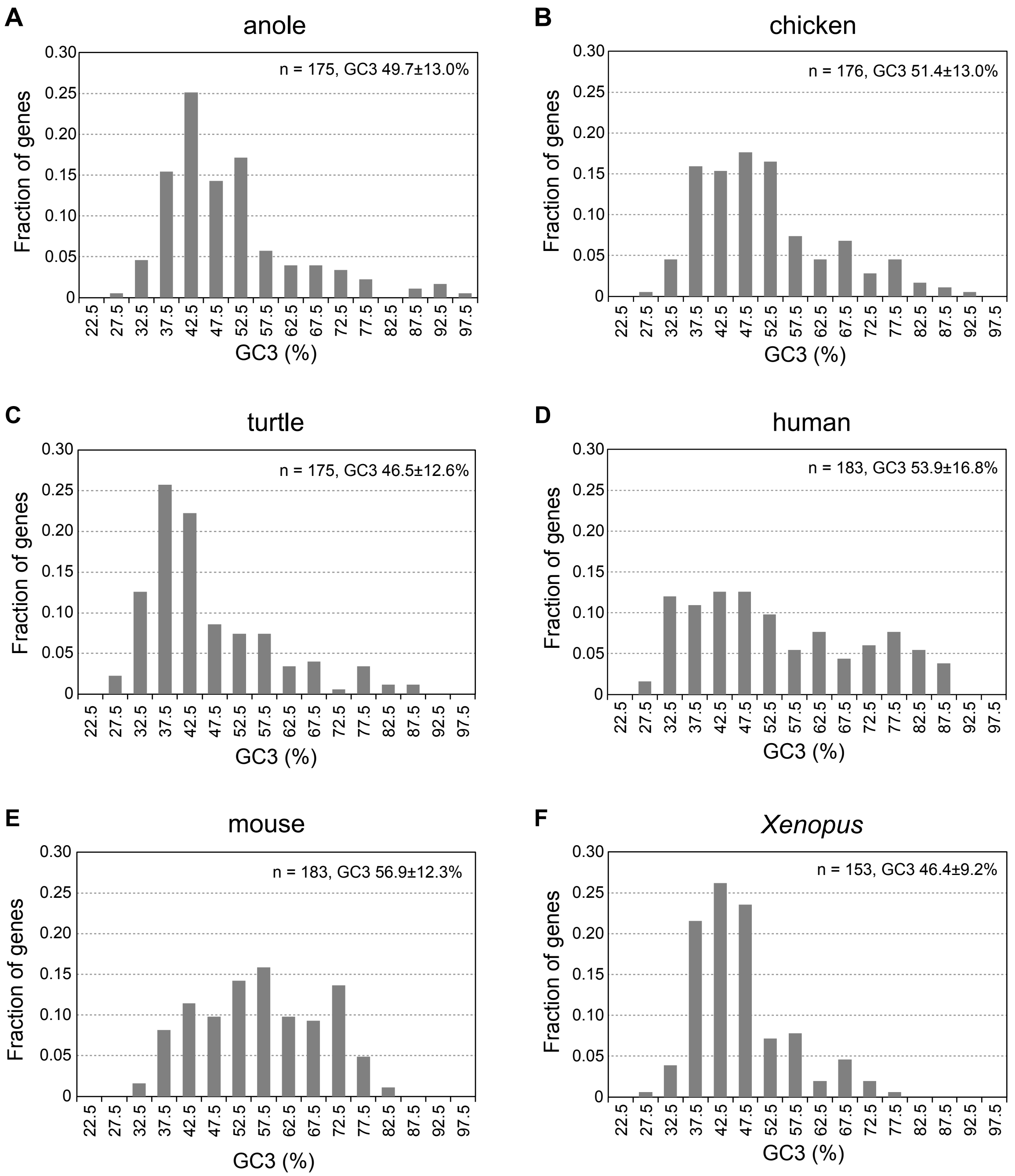

Additional file 4. GC_3 distribution of the orthologs in other vertebrates. Histograms show frequency distributions of orthologs in green anole lizard (A), chicken (B), Chinese soft-shelled turtle (C), human (D), mouse (E) and *Xenopus tropicalis* (F).
